# Fatou Components of Attracting Skew-Products

**DOI:** 10.1007/s12220-017-9811-6

**Published:** 2017-04-06

**Authors:** Han Peters, Iris Marjan Smit

**Affiliations:** KdV Institute for Mathematics, University of Amsterdam, Amsterdam, The Netherlands

**Keywords:** Holomorphic, Dynamics, Complex, 32H

## Abstract

We investigate the existence of wandering Fatou components for polynomial skew-products in two complex variables. In 2004, the non-existence of wandering domains near a super-attracting invariant fiber was shown in Lilov (Fatou theory in two dimensions, PhD thesis, University of Michigan, 2004). In 2014, it was shown in Astorg et al. (Ann Math, arXiv:1411.1188 [math.DS], 2014) that wandering domains can exist near a parabolic invariant fiber. In Peters and Vivas (Math Z, arXiv:1408.0498, 2014), the geometrically attracting case was studied, and we continue this study here. We prove the non-existence of wandering domains for subhyperbolic attracting skew-products; this class contains the maps studied in Peters and Vivas (Math Z, arXiv:1408.0498, 2014). Using expansion properties on the Julia set in the invariant fiber, we prove bounds on the rate of escape of critical orbits in almost all fibers. Our main tool in describing these critical orbits is a possibly singular linearization map of unstable manifolds.

## Introduction

Sullivan’s No Wandering Domains Theorem [10] states that every Fatou component of a rational function $f:\hat{\mathbb C} \rightarrow \hat{{\mathbb {C}}}$ is either periodic or pre-periodic. It was recently shown in [1] by Astorg et al. and the first author that Sullivan’s theorem does not hold for polynomial maps in ${\mathbb {C}}^2$. The maps constructed in [1] are polynomial skew-products of the form

$$\begin{aligned} (z,w) \mapsto \bigl (f(z,w), g(w)\bigr ), \end{aligned}$$

where $f(z,w) = p(z) + q(w)$ and both polynomials *p* and *g* have a parabolic fixed point at the origin. The wandering Fatou components are contained in the parabolic basin of *g*.

In the current project, we investigate whether polynomial skew-products can also have wandering domains in the basin of an attracting fixed point of *g*. Throughout the paper, we will assume that the first coordinate function *f* of the skew-product has the form $f(z,w) = z^d + a_{d-1}(w) z^{d-1} + \cdots + a_0(w)$. Recall that in the paper on polynomial skew-products by Jonsson [4], a polynomial skew-product is assumed to extend holomorphically to ${\mathbb {P}}^2$. We do not make this stronger assumption here. We will prove the following.

### Theorem 1

Let $F:(z,w)\mapsto (f(z,w),g(w))$ be a polynomial skew-product and assume that $0 = g(0)$ is an attracting fixed point with corresponding basin $B_g$. Further assume that the polynomial $p(z) = f(z,0)$ is subhyperbolic. Then *F* has no wandering Fatou components contained in ${\mathbb {C}} \times B_g$.

The notion of subhyperbolicity will be discussed later in this introduction. Due to the skew-product form of the polynomial map *F*, we may identify a Fatou component *U* of *g* with the open subset $({\mathbb {C}} \times U) \subset {\mathbb {C}}^2$. For example, we may refer to ${\mathbb {C}} \times B_g$ as the attracting basin of the polynomial *g*.

Using this terminology, we note that any Fatou component of a skew-product *F* must be contained in a Fatou component of the polynomial *g*. Hence if *F* has a wandering Fatou component, then by Sullivan’s Theorem *F* must have a wandering Fatou component that is contained in a periodic Fatou component of *g*. This periodic component must be either attracting, parabolic or a Siegel disk. As mentioned before, the existence of wandering domains in parabolic basins of *g* was shown in [1]. In [7], Lilov showed that there can be no wandering Fatou components in a super-attracting basin of *g*. In this work, we will focus on the geometrically attracting case.

Without loss of generality, we may assume that $0 = g(0)$ is an attracting fixed point. Note that if we can rule out the existence of wandering domains in a small neighborhood of $\{w=0\}$, then it follows that there are no wandering domains in the entire basin of attraction of 0. When working in a small neighborhood of $\{w=0\}$, we can change coordinates so that $g(w) = \lambda w$, with $0< |\lambda | < 1$. A consequence is that the coefficients $a_0, \ldots a_{d-1}$ of *f* are generally no longer polynomials, but only depend holomorphically on *w*. This will not be an issue for us, and in fact our results hold for *g* holomorphic as well. For simplicity of notation, we will continue to assume that $F(z,w) = (f(z,w) , \lambda w)$ is a polynomial skew-product.

The geometrically attracting case was recently studied by Vivas and the first author in [8]. In order to state results from that paper, let us first recall in greater detail what Lilov proved for the super-attracting case. Lilov first showed that every 1-dimensional Fatou component of $p(\cdot ) = f(\cdot ,0)$ is contained in a Fatou component of *F*, which we will refer to as a *bulging* Fatou component of *p* (by slight abuse of language). Then, Lilov showed that the forward orbit of any nearby horizontal disk must intersect a bulging Fatou component of *p*. By horizontal disk, we mean a disk contained in a fiber $\{w = w_0\}$, and by nearby we mean that $w_0$ is contained in the basin of $\{w = 0\}$. The non-existence of wandering Fatou components in a super-attracting basin of *g* follows immediately.

The bulging of Fatou components holds in the geometrically attracting case as well. However, in [8], explicit attracting polynomial skew-products were constructed for which there are nearby horizontal disks whose forward orbits avoid the bulging Fatou components of *p*. What we show in the current work is that in *most* fibers such disks cannot exist.

### Proposition 2

Consider a skew-product

$$\begin{aligned} F(z,w) = \bigl (f(z,w), \lambda w\bigr ) \end{aligned}$$

satisfying the conditions of Theorem 1, with $0<|\lambda |<1$. Then there is a set $E \subset {\mathbb {C}}$ of full Lebesgue measure, such that for every $w_0 \in E$ the forward orbit of every disk in the fiber $\{w = w_0\}$ must intersect a bulging Fatou component of *p*.

In particular the set *E* is dense, so Theorem 1 follows immediately. Another stronger corollary is that the only Fatou components of *F* are the bulging Fatou components of *p*. This follows immediately from the fact that the topological degree of *F* equals the degree of *p*, and hence the only Fatou components that can be mapped onto the bulging Fatou components of *p* are those bulging Fatou components.

The skew-products constructed in [8] are of the form

$$\begin{aligned} F(z,w) = \bigl (p(z) + q(w), \lambda w\bigr ), \end{aligned}$$

where *p* has only a single critical point which lies in the Julia set and is pre-periodic. Hence the maps from [8] satisfy the conditions in Theorem 1.

Let us emphasize here that the proof of Sullivan’s No Wandering Domains Theorem relies in an essential way on the Mapping Theorem for quasiconformal maps. There is no known analogue for the Mapping theorem that can be used to prove the non-existence of wandering domains in higher dimensions. In light of the construction of wandering domains in [1], it seems unlikely that this line of argument can be used even for polynomial skew-products. For general one-dimensional polynomial or rational functions, there is no alternative for the use of quasiconformal deformations to prove the non-existence of wandering domains.

However, under additional assumptions on the post-critical set, there do exist alternative proofs. The easiest class is given by the hyperbolic polynomials, for which the forward orbits of all critical points stay bounded away from the Julia set. In this case, a sufficiently large iterate of the map is expanding on the Julia set, which immediately implies the non-existence of wandering Fatou components. It was pointed out by Lilov [7] that an attracting or super-attracting skew-product acting hyperbolically on the invariant fiber has no wandering Fatou components. In this article, we consider skew-products satisfying a weaker assumption, namely that the polynomial acting on the invariant fiber is subhyperbolic.

In the subhyperbolic case, sometimes referred to as Misiurewicz, the polynomial is assumed to have no parabolic periodic points, and the critical points that lie on the Julia set are pre-periodic. In fact, these assumptions can be taken as the definition of subhyperbolicity, an alternative definition being that the polynomial is expanding with respect to a so-called admissible metric.

Let us recall a classical argument due to Fatou to show that a subhyperbolic polynomial *p* does not have wandering Fatou components. Imagine that it does, and let *K* be a compact subset of such a wandering Fatou component. Then for some subsequence $(n_j)$ the sets $f^{n_j}(K)$ must converge to a point $x \in \mathcal {J}_p$ that does not lie in the post-critical set. Let *U* be a disk centered at *x* that does not intersect the post-critical set. Then on *U* all inverse branches of every iterate $p^n$ are conformal, and necessarily map *U* inside the disk of radius *R*, the escape radius. It follows that as the Poincaré diameter of $f^{n_j}(K)$ in *U* converges to 0, the Poincaré diameter of *K* in $D_0(R)$ must also converge to 0, which gives a contradiction.

We will follow the same line of argument in this paper, the main difference being that instead of having no critical points enter the disk *U*, we obtain sub-linear estimates on the degree of the inverse branches. This will turn out to be sufficient to conclude the non-existence of wandering domains.

The outline of the proof of Proposition 2 is discussed in the next section. The details are covered in Sects. 3–7. In Sect. 4, we discuss the proof under stronger assumptions, close to the assumptions used in [8], in which case a much shorter argument is obtained.

## Outline and Background

Throughout the rest of this paper, we work with a map

$$\begin{aligned} F(z,w)=\bigl (f(z,w),\lambda w\bigr ), \end{aligned}$$

where $|\lambda |<1, f(z,w)=z^d+a_{d-1}(w)z^{d-1}+\cdots +a_0(w)$ and $p(z)=f(z,0)$. We will write $F^n$ for the *n*th iterate of *F*, and we will use the convenient notation

$$\begin{aligned} F^n(z,w) = \bigl (F^n_1(z,w), F^n_2(z,w)\bigr ). \end{aligned}$$

We assume that the polynomial *p* is subhyperbolic. A consequence is that the Fatou set consists of a finite collection of attracting basins, and that the orbit of each critical point in the Fatou set converges to one of the attracting cycles. Every critical point in $\mathcal {J}_p$ is eventually mapped onto a repelling periodic point. Passing to some iterate of *F* and *p*, we may assume that all our critical points in $\mathcal {J}_p$ are not just pre-periodic, but will eventually be mapped onto repelling fixed points.

A subhyperbolic polynomial is in particular also *semi-hyperbolic*. Recall that a polynomial *p* is called semi-hyperbolic if the Julia set does not contain parabolic periodic points or recurrent critical points. It is an interesting question whether the methods introduced in this article can be used to prove Theorem 1 under the more general assumption that *p* is semi-hyperbolic.

The main idea in the proof of Proposition 2 is that the fibers $\{w = w_0\}$ in *E* are chosen such that the critical points in the fibers $\{w = \lambda ^n w_0\}$ escape a given neighborhood of the Julia set sufficiently fast under iterations of *F*. To be more precise, fix $R>0$ large enough such that

$$\begin{aligned} |z|> R \; \; \hbox {implies that} \; \; |p(z)| > 2|z|. \end{aligned}$$

For each attracting periodic point *y* of *p*, fix an open neighborhood $W_y$ of its orbit such that $\overline{p(W_y)} \subset W_y$. Define

$$\begin{aligned} W_0=\{|z|>R\} \cup \bigcup _{y}W_y, \end{aligned}$$

where we take the union over all attracting periodic points $y \in {\mathbb {C}}$.

Since *p* has only finitely many attracting periodic orbits, we can find $\epsilon >0$ such that the set

$$\begin{aligned} W:=W_0 \times D(0,\epsilon )\subseteq \mathbb {C}^2 \end{aligned}$$

satisfies $\overline{F(W)} \subseteq W$. Fix a constant *M* such that $|DF| \le M$ on $D(0,R+1) \times D(0,\epsilon )$.

Define the sets $U_m=p^{-m}(W_0)$ and write $V_m$ for their complements. By slight abuse of notation we consider these as subsets of $\mathbb {C}_z = \mathbb {C} \times \{0\} \subset {\mathbb {C}}^2$ as well. Notice that

$$\begin{aligned} \mathcal {F}_p = \bigcup _{m \in \mathbb {N}} U_m. \end{aligned}$$

By definition, points in $U_m$ must escape to $W_0$ in at most *m* steps. An elementary computation shows that a similar statement holds for points sufficiently close to $U_m$:

### Lemma 3

There exist $C_1>0$ such that for all $m \in {\mathbb {N}}$ the following holds:

$$\begin{aligned} \text {If }\text {d}\bigl ((z,w),U_m \bigr ) < C_1 M^{-m}, \quad \text {then }F^{m+1}(z,w) \in W. \end{aligned}$$

Since *p* is subhyperbolic, it is also semi-hyperbolic. These polynomials were studied by Carleson et al. [2]. In Sect. 5, we will use their estimates to prove the following:

**Proposition** 10. * The area of the sets* $V_m$ *decreases exponentially with* *m*.

We note that this estimate does not hold for general polynomials; for example, it does not hold for polynomials with a parabolic periodic point. As the exponential decay of these areas will play a crucial role in our proof, it is clear that one should not expect our proof to work for polynomials *p* that are not semi-hyperbolic.

Let $U\subseteq \mathbb {C}$ be any neighborhood of the post-critical set of *p*. Using Proposition 10, we will be able to choose fibers $\{w = w_0\}$ for which all critical points in the fibers $\{w = \lambda ^n w_0\}$ escape to *W*, with bounds on the number of steps it takes for the orbit of such a critical point to leave $U \times \mathbb {C}$ and land in *W*. As a consequence we will obtain the following:

**Proposition** 15. *There exists a set* $E \subset \mathbb {C}$, *of full measure in some neighborhood of the origin, with the following property: For all* $w \in E$ *there exists a constant* $C_2 = C_2(w,U)$ *such that for all* $n \in \mathbb {N}$ *we have*

$$\begin{aligned} \#\left\{ z:~ \frac{\partial F_1^n}{\partial z}(z,w)=0 \mathrm {\; and \;} F_1^n(z,w) \notin W_0 \cup U \right\} \le C_2 \sqrt{n}. \end{aligned}$$

Recall that each critical point $x \in \mathcal {J}_p$ of *p* is eventually mapped to a repelling fixed point $x_r$, with multiplier $\mu$ where $|\mu |>1$. The main tool for controlling the orbits of the critical points of the polynomial skew-product is a linearization map of the unstable manifold of this repelling fixed point, given by a map $\varPhi :{\mathbb {C}} \rightarrow {\mathbb {C}}$ that satisfies $\varPhi (\mu t) = p^k \circ \varPhi (t)$ for some $k \in \mathbb {N}$. The construction of these linearization maps will be discussed in Sect. 3.

The bound on the number of critical points provided by Proposition 15 gives us degree estimates for some proper holomorphic maps between hyperbolic Riemann surfaces, which in turn can be used to obtain area-estimates, using the following proposition, which will be proved in Sect. 7.

**Proposition** 28. * There exists a uniform constant* $C_3>0$ *such that the following holds: Let* $f:{\mathbb {D}} \rightarrow {\mathbb {D}}$ *be a proper holomorphic map of degree* *d*, *and let* $R \subseteq \mathbb {D}$ *have Poincaré area* *A*. *If* $d\cdot A^{1/2d}<1/8$, *then the Poincaré area of* $f^{-1}(R)$ *will be at most* $C_3 d^3 A^{1/d}$.

With these ingredients we are ready to prove our main results.

### Proof of Proposition 2

Let *E* be as in Proposition 15, and suppose for the purpose of a contradiction that a fiber $\{w = w_0\}$, where $w_0 \in E$, does contain a disk *D* whose forward orbit avoids the bulging Fatou components of *p*. Then the restriction of $F^n$ to *D* is bounded and hence a normal family. Therefore, there exists a subsequence $F^{n_j}$ such that $F^{n_j}|_D$ converges, uniformly on compact subsets of *D*, to a holomorphic map to $\mathcal {J}_p$. As $\mathcal {J}_p$ has no interior, $F^{n_j}(D)$ must in fact converge to a point $\zeta \in \mathcal {J}_p$. After shrinking *D* if necessary, we may assume that the convergence of $F^{n_j}(D)$ to $\zeta$ is uniform.

In particular, as $F(W) \subseteq W$, we know that $F^n(D)$ will never intersect *W*. It therefore follows from Lemma 3 that whenever $|\lambda ^n w_0| < C_1 M^{-m}$, we have $F_1^n(D) \subseteq V_m$. By Proposition 10, we know that $\text {Area}(V_m)$ decreases exponentially. Hence there exist constants $C > 1$, and $\gamma < 1$ such that

1 $$\begin{aligned} \mathrm {Area}(F^n(D)) \le C \gamma ^n. \end{aligned}$$

By our assumption that the critical points in $\mathcal {J}_p$ are all eventually mapped onto repelling fixed points, we may assume that $\zeta$ does not lie in the post-critical set. So choose our open neighborhood $U \subseteq \mathbb {C}_z$ of the post-critical set in such a way that $\zeta \notin U$, and let $r>0$ be such that $D(\zeta ,r) \cap (U \cup W_0)= \emptyset$. Let $J_1 \in {\mathbb {N}}$ be such that $F_1^{n_j}(D) \subseteq D(\zeta ,\frac{r}{2})$ for all $j \ge J_1$. Define $O_j$ to be the connected component of $\left( F^{n_j} \right) ^{-1}\left( D(\zeta ,r)\times \{\lambda ^{n_j} w_0\}\right)$ that contains *D*. Then we have $D \subseteq O_j \subseteq D(0,R)\times \{w_0\}$, and we can regard $F_1^{n_j} :O_j \rightarrow D(\zeta ,r)$ as a one-dimensional proper holomorphic map.

Proposition 15 tells us that this map has at most $d_j=C_2\sqrt{n_j}$ critical points. We are now in position to apply Proposition 28. Set $R_j=F_1^{n_j}(D)$, with Poincaré area $A_j=\text {Area}_{D(\zeta ,r)}(R_j)$ with respect to $D(\zeta ,r)$. As $j \ge J_1$, we have $R_j\subseteq D(\zeta ,\frac{r}{2})$. Hence we can estimate $A_j$ using our result on the Euclidean area of $R_j$ as found in Eq. (1): $A_j<C'\gamma ^{n_j}$ for some uniform constant $C'$. Then

$$\begin{aligned} d_j A_j^{1/2d_j}< C_2 \sqrt{n_j} \left( C' \gamma ^{n_j} \right) ^{1/2C_2 \sqrt{n_j}}<C_2(C')^{1/2C_2 \sqrt{n_j}} \sqrt{n_j} \gamma ^{\sqrt{n_j}/2C_2}. \end{aligned}$$

Since $\gamma <1$, this expression converges to zero as *j* increases. Therefore, we can find $J_2>J_1$ such that $d_j A_j^{1/2d_j}<1/8$ for all $j>J_2$.

In this setting, Proposition 28 implies that

$$\begin{aligned} \text {Area}_{D(0,R)}(D) \le \text {Area}_{O_j}(D) \le C_3 d_j^3 A_j^{1/d_j} < C_3C_2^3 n_j^{3/2} \left( C' \gamma ^{n_j} \right) ^{1/C_2 \sqrt{n_j}}. \end{aligned}$$

However, this last expression will also tend to zero as *j* increases, which gives a contradiction. $\square$

For the rest of this paper, our goal is to prove Propositions 10, 15, and 28. First, we define the linearization map $\varPhi$ that is used to describe the behavior of the critical points in the Julia set. In Sect. 4, we prove our results for the special class of maps studied by Vivas and the first author in [8]. In this setting, the proof is much shorter, and we will give a more precise description of the post-critical orbits.

In Sect. 5, we get back to the general case and prove Proposition 10. This estimate will be used to prove Proposition 15 in the next section. And finally, we prove Proposition 28 in Sect. 7.

## Linearization Maps

In this section, we construct the linearization map $\varPhi$ that will later help us track the orbits of the critical points of *F*. As $F(z,w)=\left( f(z,w), \lambda w\right)$ with $f(z,w)= z^d+a_{d-1}(w)z^{d-1}+ \cdots + a_0(w)$, the critical points of *F* are those points where $\frac{\partial f(z,w)}{\partial z}=0$.

In a small neighborhood $\{|w|<\epsilon \}$ of the $\{w=0\}$-fiber, these critical points will form finitely many, possibly singular, varieties $K_1, \ldots , K_q$, each of which intersects $\{w=0\}$ in a single point. Let *K* be such a variety, with intersection $(x_0,0)$ with $\{w=0\}$. If $x_0 \in \mathcal {J}_p$, then $x_0$ must be eventually mapped to a repelling fixed point $x_r=p^r(x_0)$ of *p*, which is a saddle point for *F*. The variety $F^r(K)$ will pass through this saddle point. The linearization results in this section will help us study the orbits originating on these varieties.

Let $G:({\mathbb {C}}^2,0) \rightarrow ({\mathbb {C}}^2,0)$ be a holomorphic germ, and assume that 0 is a saddle fixed point. Without loss of generality, we may assume that the stable direction is (0, 1) and the unstable direction is (1, 0), so that *G* is of the form

$$\begin{aligned} G:(z,w) \mapsto (\mu z + h.o.t., \lambda w + h.o.t.), \end{aligned}$$

with $|\mu |>1$ and $|\lambda | < 1$. After changing coordinates, we may assume that, possibly on a smaller neighborhood of the origin, the stable and unstable manifolds are equal to respectively ${\mathbb {C}}_w=\{0\} \times \mathbb {C}$ and ${\mathbb {C}}_z=\mathbb {C} \times \{0\}$.

Let *V* be an irreducible analytic variety through the origin. We can locally write *V* as

$$\begin{aligned} V = \{(\gamma _1(t), \gamma _2(t))\}, \end{aligned}$$

where

$$\begin{aligned} \gamma _1(t) = a t^k + h.o.t. \; \; \; \mathrm {and} \; \; \; \gamma _2(t) = b t^l + h.o.t.. \end{aligned}$$

### Remark 4

When $\gamma _1=0$, the variety *V* coincides with the stable variety $\{z=0\}$ of *G*. In this case, $(G^n(\gamma (t)))_{n \in \mathbb {N}}$ will converge to the origin along the line $\{z=0\}$, uniformly on compact subsets.

When $\gamma _1 \ne 0$, the order *k* of the parametrization is well defined. We will prove the following proposition:

### Proposition 5

The maps $\varPhi _n$ defined by

$$\begin{aligned} \varPhi _n(t) = G^{kn}(\gamma _1(\mu ^{-n}t), \gamma _2(\mu ^{-n}t)) \end{aligned}$$

converge uniformly on compact subsets of ${\mathbb {C}}$ to a holomorphic map

$$\begin{aligned} \varPhi :{\mathbb {C}} \rightarrow {\mathbb {C}}_z \end{aligned}$$

of local order *k* that satisfies the functional equation $G^k\circ \varPhi (t) = \varPhi (\mu t)$. This convergence is exponentially fast on compact subsets.

### Proof

Let us start with the case where $\gamma _2=0$, as it is more straightforward. We use the following variation on Koenigs’ theorem.

### Lemma 6

Let

$$\begin{aligned} g(z) = \mu ^k z + h.o.t. \end{aligned}$$

and

$$\begin{aligned} h(t) =a t^k + h.o.t. \end{aligned}$$

be holomorphic functions defined in a neighborhood of the origin, with $k \ge 1, a \ne 0$ and $|\mu | > 1$. Then the sequence of maps

$$\begin{aligned} \varphi _n(t) = g^n \circ h(\mu ^{-n} t) \end{aligned}$$

converges uniformly and exponentially fast on compact subsets of ${\mathbb {C}}$.

### Proof

Using Koenigs’ theorem we can write

$$\begin{aligned} g(z) = \psi ^{-1}(\mu ^k \cdot \psi (z)), \end{aligned}$$

for a germ $\psi$ of the form

$$\begin{aligned} \psi (z) = z + h.o.t. \end{aligned}$$

Hence $$g^n(z) = \psi ^{-1} ( \mu ^{kn} \psi (z))$$, and

$$\begin{aligned} g^n \circ h(\mu ^{-n} t) = \psi ^{-1}\left( \mu ^{kn} \psi \circ h(\mu ^{-n}t)\right) . \end{aligned}$$

Writing

$$\begin{aligned} \psi \circ h(t) = a t^k + t^{k+1} \cdot \eta (t), \end{aligned}$$

we obtain

$$\begin{aligned} \mu ^{k(n+1)} \psi \circ h (\mu ^{-(n+1)}t) - \mu ^{kn} \psi \circ h (\mu ^{-n}t) = \mu ^{-n}t^{k+1}\left[ \mu ^{-1}\eta (\mu ^{-(n+1)}t)-\eta (\mu ^{-n}t)\right] . \end{aligned}$$

The statement follows. $\square$

We continue the proof of Proposition 5. Use the notation

$$\begin{aligned} G^k(z,w)=\left( G_1^k(z,w),G_2^k(z,w)\right) \end{aligned}$$

for the components of the *k*th iterate of *G*. Since *G* leaves both axes invariant, we can write

$$\begin{aligned} G_1^k(z,w)=\mu ^k z + z \cdot f_1(z,w) \quad \text { and } \quad G_2^k(z,w)=\lambda ^k w + w \cdot f_2(z,w), \end{aligned}$$

where $$f_1(0,0) = f_2(0,0) = 0$$.

By our assumption that $\gamma _2=0$, it follows that $\varPhi _n(t) = \left( g^{n}(\gamma _1(\mu ^{-n} t)),0\right)$ for all $n \in \mathbb N$, where $g(\cdot )=G_1^k(\cdot ,0)$. By Lemma 6 the sequence $g^{n}(\gamma _1(\mu ^{-n} t))$ converges uniformly and exponentially fast on compact subsets of ${\mathbb {C}}$.

Now that we have dealt with the cases where $\gamma _1=0$ or $\gamma _2=0$, we tackle the general case. Write $\gamma _1(t)=a t^k + t^{k+1} {\tilde{\gamma }}_1(t)$ with $a \ne 0$, and likewise $\gamma _2(t)=b t^l + t^{l+1} {\tilde{\gamma }}_2(t)$ with $b \ne 0$.

Define

$$\begin{aligned} \varPhi _n(\zeta , \xi ) = G^{kn}\left( \gamma _1(\mu ^{-n}\zeta ), \gamma _2(\mu ^{-n}\xi )\right) , \end{aligned}$$

as a function of two distinct variables. Our goal is to show that for $\zeta , \xi$ small we have $\varPhi _{n}(\zeta , \xi ) = \varPhi _{n-1}(\zeta ^\prime , \xi ^\prime )$, where $\zeta ^\prime \sim \zeta$ and $|\xi ^\prime | \ll |\xi |$.

We start with the trivial equation

$$\begin{aligned} \varPhi _{n}(\zeta , \xi ) = G^{k(n-1)} \circ G^k \left( \gamma _1(\mu ^{-n}\zeta ), \gamma _2(\mu ^{-n}\xi )\right) . \end{aligned}$$

To unravel this, note that for $\zeta , \xi \in D(0,\epsilon )$ with $\epsilon$ sufficiently small (independent of *n*), we have

$$\begin{aligned} G_1^k&\left( \gamma _1(\mu ^{-n}\zeta ),\gamma _2(\mu ^{-n}\xi )\right) \\&= a \mu ^{-k(n-1)}\zeta ^k + \mu ^{-k(n-1)-n} \zeta ^{k+1}{\tilde{\gamma }}_1(\mu ^{-n}\zeta )\\&\quad +\,\gamma _1(\mu ^{-n}\zeta )f_1\left( \gamma _1(\mu ^{-n} \zeta ),\gamma _2(\mu ^{-n}\xi )\right) \\&= a (\mu ^{-(n-1)} \zeta )^k \biggl (1+ \mu ^{-n} a^{-1} \zeta {\tilde{\gamma }}_1(\mu ^{-n}\zeta ) \\&\quad +\,\mu ^{-n}a^{-1}\mu ^{-k} \frac{\gamma _1(\mu ^{-n}\zeta )}{(\mu ^{-n}\zeta )^k}\frac{f_1\left( \gamma _1(\mu ^{-n} \zeta ),\gamma _2(\mu ^{-n}\xi )\right) }{\mu ^{-n}}\biggr )\\&=\gamma _1\left( \mu ^{-(n-1)} \psi _{n}(\zeta ,\xi )\right) . \end{aligned}$$

Here, the function $\psi _{n}$ has the form $\psi _{n}(\zeta ,\xi )=\zeta (1+\mu ^{-n}{\tilde{\psi }}_{n}(\zeta ,\xi ))$ with ${\tilde{\psi }}_{n}$ holomorphic and bounded on $D(0,\epsilon ) \times D(0,\epsilon )$, uniformly in *n*.

Likewise, we can work out that

$$\begin{aligned} G_2^k&\left( \gamma _1(\mu ^{-n}\zeta ),\gamma _2(\mu ^{-n}\xi )\right) \\&=\lambda ^k b \mu ^{-ln} \xi ^l +\lambda ^k \mu ^{-nl-n}\xi ^{l+1}{\tilde{\gamma }}_2(\mu ^{-n}\xi ) +\gamma _2(\mu ^{-n}\xi )f_2\left( \gamma _1(\mu ^{-n} \zeta ),\gamma _2(\mu ^{-n}\xi )\right) \\&=b_k (\lambda ^{k/l}\mu ^{-n}\xi )^l \biggl (1+ \mu ^{-n}b^{-1}\xi {\tilde{\gamma }}_2(\mu ^{-n}\xi )\\&\qquad + \mu ^{-n}b_k^{-1}\lambda ^{-k} \frac{ \gamma _2(\mu ^{-n}\xi )}{(\mu ^{-n}\xi )^l}\frac{f_2\left( \gamma _1(\mu ^{-n}\zeta ),\gamma _2(\mu ^{-n}\xi )\right) }{\mu ^{-n}}\biggr )\\&=\gamma _2\left( \mu ^{-(n-1)}\chi _n(\zeta ,\xi )\right) . \end{aligned}$$

The function $\chi _n$ has the shape $\chi _n(\zeta ,\xi )=\lambda ^{k/l}\mu ^{-1} \xi \bigl (1+\mu ^{-n} {\tilde{\chi }}_n(\zeta ,\xi )\bigr )$, once again with ${\tilde{\chi }}_{n}$ bounded on $D(0,\epsilon ) \times D(0,\epsilon )$, uniformly in *n*.

Writing $\varphi _n=(\psi _n,\chi _n)$, we now have

$$\begin{aligned} \varPhi _n(\zeta ,\xi )=\varPhi _{n-1} \circ \varphi _n(\zeta ,\xi ). \end{aligned}$$

Let *C* be an upper bound for all $|{\tilde{\psi }}_n|$ and $|{\tilde{\chi }}_n|$ on $D(0,\epsilon )\times D(0,\epsilon )$, and choose *N* large enough to ensure that $|\frac{\mu ^{-N}C}{1-|\mu ^{-1}|}| < \epsilon /2$ and $|\lambda ^{k/l}\mu ^{-1} |(1+|\mu |^{-N} C)<1$. Then a straightforward induction on *m* shows that

$$\begin{aligned} \varphi _{n}\circ \cdots \circ \varphi _{n+m}(\zeta , \xi ) \in D\left( 0,\frac{\epsilon }{2}+\frac{ |\mu |^{-n}C}{1-|\mu |^{-1}}\right) \times D(0, \epsilon ) \end{aligned}$$

for all $n \ge N, m \in \mathbb {N}$ and $(\zeta ,\xi ) \in D(0,\epsilon /2)\times D(0,\epsilon )$.

We can now write $\varPhi _n(\zeta ,\xi )=\varPhi _N\left( \varphi _{N+1} \circ \cdots \circ \varphi _n(\zeta ,\xi )\right)$. By our bounds on $|{\tilde{\psi }}_n|$ and $|{\tilde{\chi }}_n|$, the sequence $(\varPhi _n(\zeta ,\xi ))_{n \ge N}$ will converge uniformly and exponentially fast on $D(0,\epsilon /2)\times D(0,\epsilon )$.

Plugging in $\zeta =\xi =t$ now proves the proposition: $(\varPhi _n(t))_{n \in \mathbb {N}}$ must converge on $D(0,\epsilon /2)$. By construction, it must satisfy the functional equation

$$\begin{aligned} G^k \circ \varPhi (t)=\varPhi (\mu t). \end{aligned}$$

This equation also gives us global convergence of $(\varPhi _n(t))_{n \in \mathbb {N}}$ and uniform exponentially fast convergence on compact subsets. By construction, $\varPhi$ must have local order *k*. $\square$

### Remark 7

Let us say a few words about the role that the linearization map $\varPhi$ will play in the rest of this paper. We have a polynomial skew-product *F* of the form

$$\begin{aligned} F(z,w) = \bigl (f(z,w), \lambda \cdot w\bigr ), \end{aligned}$$

where $f(z,w)=z^d+a_{d-1}(w)z^{d-1}+\cdots +a_0(w)$, and we write $p(\cdot ) = f(\cdot ,0)$. As mentioned at the start of this section, in some small neighborhood $\{|w|<\epsilon \}$ of the $\{w=0\}$-fiber, the critical points of *F* form finitely many irreducible critical varieties $K_1, \ldots K_q$. We may assume, shrinking $\epsilon$ if necessary, that each $K_i$ intersects $\{w=0\}$ at one unique point, which must be a critical point of *p*. Assume for now that this critical point lies in $\mathcal {J}_p$.

We study these varieties one at a time. Write *K* for our irreducible critical variety, and $x_0$ for its intersection with $\{w=0\}$. We can locally parameterize *K* by

$$\begin{aligned} K = \bigl \{(\gamma _1(t), \gamma _2(t))\bigr \}. \end{aligned}$$

By our assumptions on *p*, each of its critical points in $\mathcal {J}_p$ is eventually mapped to a repelling fixed point $p^r(x_0) = x_r$ of *p*. Write $\mu =p'(x_r)$.

Then $F^r(K)$ is a variety though $(x_r,0)$, where *F* has a saddle fixed point. Let $\varPsi$ be a local change of coordinates such that $G=\varPsi \circ F \circ \varPsi ^{-1}$ satisfies the conditions of Proposition 5: its stable and unstable manifolds are $\mathbb {C}_w$ and $\mathbb {C}_z$, respectively. We can write *G*(*z*, *w*) as

$$\begin{aligned} G :(z,w) \mapsto \bigl (\mu z+zg_1(z,w),\lambda w+wg_2(z,w) \bigr ). \end{aligned}$$

Translating to our new coordinates, our variety of interest becomes $\varPsi \left( F^r(K)\right)$. It can locally be parameterized by

$$\begin{aligned} \bigl \{\varPsi \left( F^r\left( \gamma (t)\right) \right) \bigr \}. \end{aligned}$$

If it is equal to $\mathbb {C}_w$, the original variety $F^r(K)$ must lie in the stable manifold $\Sigma _F^s(x_r)$. Otherwise, the first coordinate of $\varPsi \left( F^r\left( \gamma (t)\right) \right)$ can be written as $at^k + h.o.t.$ where $a \ne 0$ and $k \ge 1$.

Note that $\varPsi (F^{kr}(\gamma (t)))=G^{kr-r}\circ \varPsi \left( F^r\left( \gamma (t)\right) \right)$ has $\mu ^{kr-r}a t^k + h.o.t.$ as its first coordinate; the value *k* did not change. We can therefore apply Proposition 5 to find that the maps

$$\begin{aligned} \varPhi _n(t)&=F^{kn}\left( \gamma (\mu ^{-n}t)\right) \\&=\varPsi ^{-1} \circ G^{k(n-r)}\left( \varPsi \circ F^{kr} \circ \gamma \left( \mu ^{-r} \mu ^{-(n-r)}t\right) \right) \end{aligned}$$

must converge, uniformly and exponentially fast on compact subsets, to a holomorphic map $\varPhi :\mathbb {C} \rightarrow \varPsi ^{-1}(\mathbb {C}_z)=\Sigma _F^u(x_r)=\mathbb {C}_z$ of local order *k*. By construction, $\varPhi$ must satisfy the functional equation $F^k \circ \varPhi (t) = \varPhi (\mu t)$. This map will be used all through Sects. 4 and 6 to study the orbits of critical points in varieties *K* that pass through the Julia set of *p*.

## The Resonant Case

In this section, we consider the special resonant case, which was also studied by Vivas and the first author in [8]. In this case, it is considerably easier to prove the non-existence of wandering domains, and we will obtain a more precise description of the critical orbits. Although the results in this section are not used in the proofs of Proposition 2 and Theorem 1, they did serve as an inspiration.

We consider polynomial skew-products of the form

$$\begin{aligned} F(z,w) = \bigl (p(z) + q(w), \lambda \cdot w\bigr ), \end{aligned}$$

for which we now assume that the polynomial *p* has only a single critical point $x_0$, which in a finite number of steps is mapped onto a repelling fixed point $x_r = p^r(x_0)$. Without loss of generality, we may assume that $x_r = 0$. As usual we write $p^\prime (0) = \mu$, which satisfies $|\mu |>1$. To keep the study case in this section as straightforward as possible, we assume that the critical variety $K=\{(x_0,t)\}$ has an image $F^r(K)$ that is transverse to the stable variety $\Sigma _F^s(0)$. We further assume the following resonant condition:

$$\begin{aligned} \mu \cdot \lambda = 1. \end{aligned}$$

While the resonant case is the only case for which the existence of horizontal disks that avoid the bulging Fatou components of *p* has been shown, it turns out that it is also the simplest case where we can show that most fibers $\{w = w_0\}$ contain no such disks. In fact, in this case the set *E* of such fibers will not only have full measure, but will also be open and dense.

We use the linearization function $\varPhi :\mathbb {C} \rightarrow \mathbb {C}_z$ introduced in Sect. 3, given in this resonant case by

$$\begin{aligned} \varPhi (t)=\lim _{n \rightarrow \infty } F^n\left( x_0, \lambda ^n t\right) . \end{aligned}$$

By Remark 7 and our assumption that $F^r(K)$ is transverse to $\Sigma _F^s(0)$, we know that $\varPhi$ is a non-constant holomorphic function.

### Proposition 8

Let $w_0 \in {\mathbb {C}}$ be such that $\varPhi (w_0)$ lies in the basin of infinity of the polynomial *p*. Then the orbit of any horizontal disk in the $\{w = w_0\}$ fiber must intersect the bulging Fatou component of *p*.

Note that under our assumptions the filled Julia set of *p* has empty interior, and hence the basin of infinity is open and dense. Since $\varPhi$ is a non-constant holomorphic function, it follows that the above proposition holds for an open and dense set of parameters $w_0$, and in particular that *F* does not have wandering Fatou components. The interested reader should have no difficulty generalizing Proposition 8 to the case of several critical points that are all mapped to resonant repelling periodic points.

In preparation for the proof of Proposition 8, we will study the orbits of the critical points $(x_0, \lambda ^n w_0)$ for $w_0$ such that $\varPhi (w_0)$ lies in the basin of infinity of *p*. More precisely, we will show that, for $n \in {\mathbb {N}}$ sufficiently large, the orbits of the critical points $(x_0, \lambda ^n w_0)$ will stay uniformly away from any point in $\mathcal {J}_p \setminus \{x_0, \ldots , x_r\}$.

For $n \in \mathbb {N}, m \in \mathbb {Z}$ and $n+m \ge 0$ we define

$$\begin{aligned} a_{n,m}=F^{n+m}\left( x_0, \lambda ^n w_0 \right) . \end{aligned}$$

Note that $a_{n,0}=\varPhi _n(w_0)$ and $F(a_{n,m})=a_{n,m+1}$. Since $F(\varPhi (t))=\varPhi (\tfrac{t}{\lambda })$, we have

$$\begin{aligned} \lim _{n \rightarrow \infty } a_{n,m}=\varPhi \left( \frac{w_0}{ \lambda ^m}\right) = F^m\left( \varPhi (w_0)\right) \end{aligned}$$

for $$m \ge 0$$, and

$$\begin{aligned} \lim _{n\rightarrow \infty } a_{n,-m}=\varPhi (\lambda ^m w_0) \in F^{-m}\left( \{\varPhi (w_0)\}\right) . \end{aligned}$$

Denote $a_m=\lim _{n \rightarrow \infty } a_{n,m}$. The sequence $(a_m)$ satisfies

$$\begin{aligned} \lim _{m \rightarrow -\infty } a_m = 0 \text { and } \lim _{m \rightarrow +\infty } a_m = \infty . \end{aligned}$$

Recall that $R>0$ was chosen such that $|p(z)| \ge 2 |z|$ for all $|z|>R$. Increasing *R* if necessary, we may also assume that $\mathcal {J}_p\subseteq D(0,R-1)$ and

$$\begin{aligned} |F(z,w)|> 2|z| \end{aligned}$$

for all $|z|>R$ and $|w|< |w_0|$. We then define

$$\begin{aligned} V=\left\{ (z,w)\in \mathbb {C}^2: |z| >R \mathrm {\; and \;} |w|<|w_0| \right\} . \end{aligned}$$

It follows that $F(V) \subseteq V$. Since $\lim _{m \rightarrow +\infty } a_m = \infty$ there exists an $N_1>0$ such that $|a_m|>R$ for all $m \ge N_1$. Then for any $\epsilon >0$ we can find a natural number $N_{\epsilon }$ such that

(i) $$|a_{n,m}-a_m|<\epsilon $$ whenever $$n \ge N_{\epsilon }, n+m \ge r$$ and $$m \le N_1$$.
(ii) $a_{n,m}\in V$ whenever $n \ge N_{\epsilon }$ and $m \ge N_1$.

Recall that $p^r(x_0) = 0$, which is a repelling fixed point of *p*. By continuity of *F* we also have that

2 $$\begin{aligned} \lim _{n \rightarrow \infty } a_{n,j-n}=p^j(x_0). \end{aligned}$$

for all $j \ge 0$. With this information, we can prove the following lemma.

### Lemma 9

Let $y \in \mathcal {J}_p \setminus \{x_0,p(x_0), \ldots p^{r-1}(x_0),0\}$. Then there exist $\delta =\delta (y)>0$ and $N=N(y) \in \mathbb {N}$ such that $a_{n,m} \notin {\bar{D}}(y,\delta ) \times \mathbb {C}$ for $n \ge N$ and $n+m \ge 0$.

### Proof

Note that all $a_m$ lie in the Fatou set of *p*, and are therefore not equal to *y*. Since $\lim _{m \rightarrow -\infty } a_m = 0$ and $\lim _{m \rightarrow \infty } a_m = \infty$ the points $a_m$ must in fact avoid some small disk $D(y,\delta _1)$. Setting $\epsilon =\delta = \delta _1/2$, and $N=N_{\epsilon }$, the statement follows whenever $n \ge N$ and $n+m \ge r$. Equation 2 allows us to increase *N* and shrink $\delta$ to deal with the case where $0 \le n+m <r$. $\square$

### Proof of Proposition 8

We follow the argument due to Fatou that we mentioned in the introduction. Suppose for the purpose of a contradiction that a horizontal disk $D= D(x,s) \times \{w_0\}$ converges to the Julia set of *p*. Since this Julia set has empty interior, we can find a subsequence $(n_j)$ such that $F^{n_j}(D)$ converges to a point $y \in \mathcal {J}_p$. Since $x_r = 0$ is repelling, we may assume that $y \notin \{x_0, \ldots , x_r\}$.

By Lemma 9, there exists a cylinder ${\bar{D}}(y,\delta ) \times \mathbb {C}$ that is avoided by $a_{n,m}$ whenever $n \ge N$ and $n+m \ge 0$. To use this property, let $F^N(x,w_0)=:(x',\lambda ^N w_0)$ and choose $s'>0$ such that $D'=D(x',s') \times \{\lambda ^N w_0\} \subseteq F^N(D)$. Then $F^{n_j-N}(D')$ will still converge to *y*.

For $j \in \mathbb {N}$ such that $n_j \ge N$, we consider the function

$$\begin{aligned} \psi _j=F^{n_j-N}(\cdot ,\lambda ^{N} w_0) :\mathbb {C} \rightarrow \mathbb {C} \times \{\lambda ^{n_j} w_0\}. \end{aligned}$$

Note that $D' \subseteq \psi _j^{-1}\left( D(y,\delta ) \times \{\lambda ^{n_j}w_0\}\right) \subset D(0,R) \times \{\lambda ^{N}w_0\}$ for all sufficiently large values of *j*. Let $O_j$ be the connected component of $D'$ in this inverse image. Since the points $a_{n,m}$ avoid $D(y,\delta ) \times \{\lambda ^{n_j}w_0\}$, it follows that $\psi _j|_{O_j}$ has no critical points. It must be a biholomorphism between $O_j$ and $D(y,\delta ) \times \{\lambda ^{n_j}w_0\}$, and hence it preserves the Poincaré metric. This gives us the inequality

$$\begin{aligned} \text {diam}_{D(0,R)}(D') \le \text {diam}_{O_j}(D')=\text {diam}_{D(y,\delta )}(F^{n_j-N}(D')). \end{aligned}$$

As $F^{n_j-N}(D')$ converges to *y*, its Poincaré diameter in $D(y,\delta )$ must tend to zero. This proves that $D'$ cannot exist. $\square$

## Semi-Hyperbolic Polynomials and Sublevel Sets

Let *p* be a semi-hyperbolic polynomial, with Fatou set

$$\begin{aligned} \mathcal {F}_p=I_{\infty } \cup \bigcup _{y} \Omega _p(y) \end{aligned}$$

equal to the union of the basin of infinity and the basins of attracting periodic cycles. Since *p* is semi-hyperbolic, Siegel disks and parabolic basins cannot occur. We define a set $W_0$ as in Sect. 2: Fix $R>0$ large enough such that

$$\begin{aligned} |z|> R \; \; \hbox {implies that} \; \; |p(z)| > 2|z|, \end{aligned}$$

and for each attracting periodic point *y* of *p*, fix an open neighborhood $W_y$ of its orbit such that $\overline{p(W_y)} \subset W_y$. Now set

$$\begin{aligned} W_0=\{|z|>R\} \cup \bigcup _{y}W_y, \end{aligned}$$

where we take the union over all attracting periodic points $y \in \mathbb {C}$.

Define the sets $U_m:=p^{-m}(W_0)$ and write $V_m$ for their complements. Notice that

$$\begin{aligned} \mathcal {F}_p = \bigcup _{m \in \mathbb {N}} U_m. \end{aligned}$$

We will prove the following.

### Proposition 10

The areas of the sets $V_m$ decrease exponentially with *m*.

We note that this proposition certainly does not hold for any polynomial; for example, it does not hold for the polynomial $z \mapsto z+z^2$. We leave the computation to the reader. To prove the above proposition, we will therefore make heavy use of the fact that *p* is semi-hyperbolic.

First of all, the semi-hyperbolicity of *p* allows us to use Theorem 2.1 in [2]:

### Theorem 11

There exist constants $\epsilon>0, c>0$, and $\theta <1$ such that for all $x \in \mathcal {J}_p$, we have

$$\begin{aligned} \mathrm{diam}(B_n(x,\epsilon )) \le c \theta ^n, \end{aligned}$$

where $B_n(x,\epsilon )$ is any connected component of $p^{-n}(D(x,\epsilon ))$.

As $\{z \in \mathbb {C}:~ |z| \le R \text { and } \text {d}(z,\mathcal {J}_p) \ge \epsilon \}$ is compact and contained in the increasing union $\bigcup _{m} U_m$, it must be contained in $U_{m_0}$ for some $m_0 \in \mathbb {N}$. Then for $m \ge m_0$, we know that $V_m \subseteq \{z \in \mathbb {C}:~\text {d}(z, \mathcal {J}_p)<\epsilon \}$. Let $z \in V_m$. Then $p^{m-m_0}(z) \in V_{m_0}$ and hence there exists $x \in \mathcal {J}_p$ such that $p^{m-m_0}(z) \in D(x, \epsilon )$. By Theorem 11, this implies that $\text {d}(z, \mathcal {J}_p) \le c \theta ^{m-m_0}$. It follows that the sets $V_m$ are contained in $\delta _m$-neighborhoods of $\mathcal {J}_p$, where $\delta _m$ decreases exponentially with *m*.

Recall from [2] that since *p* is semi-hyperbolic, the basin $I_{\infty }$ is a so-called John domain. For a John domain in $\mathbb {R}^m$, an upper bound for the Minkowski-dimension of its boundary is given in [6]. In our circumstances, it follows that $\mathrm {dim}_M(\mathcal {J}_p)=\mathrm {dim}_M(\partial I_{\infty }) < 2$. A quick consequence is the following.

### Lemma 12

Suppose that the sequence $(\delta _m)$ decreases exponentially fast. Then the area of the $\delta _m$-neighborhoods of $\mathcal {J}_p$ also decreases exponentially fast.

### Proof

Let $N_\epsilon$ be the number of $\epsilon$-disks needed to cover $\mathcal {J}_p$. Denote the Minkowski-dimension of $\mathcal {J}_p$ by *h*, and let $h< h^\prime < 2$. Then the $h^\prime$-dimensional measure of $\mathcal {J}_p$ is 0, hence for $\epsilon >0$ sufficiently small we have that

$$\begin{aligned} N_\epsilon < \frac{1}{\epsilon ^{h^\prime }}. \end{aligned}$$

Hence for *m* sufficiently large the $\delta _m$-neighborhood of $\mathcal {J}_p$ can be covered by $\epsilon ^{-h^\prime }=\delta _m^{-h^\prime }$ disks of radius $2 \delta _m$, which means that the area of this neighborhood is at most

$$\begin{aligned} \frac{4\pi \delta _m^2}{\delta _m^{h^\prime }} = 4\pi \delta _m^{2-h^\prime }. \end{aligned}$$

The statement follows. $\square$

### Proof of Proposition 10

Combining Lemma 12 with the fact that the sets $V_m$ are contained in $\delta _m$-neighborhoods of $\mathcal {J}_p$ with exponentially decreasing $\delta _m$ proves Proposition 10. $\square$

### Corollary 13

We have that

$$\begin{aligned} \sum _{m \in {\mathbb {N}}} \mathrm {Area}(V_m) < \infty . \end{aligned}$$

It is not reasonable to expect this corollary to hold for arbitrary polynomials, as $\mathcal {J}_p$ may have positive measure. It would be interesting to know whether $\sum _{m \in {\mathbb {N}}} \mathrm {Area}(V_m)$ is necessarily finite when $\mathcal {J}_p$ is assumed to have Hausdorff dimension strictly less than 2. Note that if the filled Julia set $K_p$ has no interior, then $V_m$ can be defined by $\{G \le \frac{1}{d^m}\}$, where *G* is the Green’s function. Leaving the setting of Green’s functions arising from complex dynamics, we note that there do exist compact subsets $K \subset {\mathbb {C}}$ with Hausdorff dimension strictly smaller than 2 for which the Green’s function $G_K$ with logarithmic pole at infinity satisfies

$$\begin{aligned} \sum _{m \in {\mathbb {N}}} \mathrm {Area} \left\{ G_K \le \frac{1}{2^m} \right\} = \infty . \end{aligned}$$

Such a set *K* can for example be constructed by taking the boundaries of a nested sequence of disks, and removing from these circles progressively smaller intervals. We thank Sławomir Kołodziej for pointing out this construction to us.

## Forcing Escape of Critical Points

In this section, we will prove the estimate in Proposition 15 as mentioned in the outline. We consider the family of forward iterates $F^n$ restricted to a fiber $\{w = w_0\}$ for a fixed $w_0 \ne 0$. Writing $w_n = \lambda ^n w_0$ and $f_n(z) = f_{w_n}(z) = f(z,w_n)$, we can consider these iterates $F^n$ as compositions of a sequence of polynomials, i.e.,

$$\begin{aligned} F^n|_{\{w = w_0\}} = f_{n-1} \circ \cdots \circ f_0. \end{aligned}$$

Every polynomial $f_{w_i}$ introduces new critical points. It turns out that by choosing $w_0$ carefully we can make sure that all these critical points escape to the set *W* defined in Sect. 2, and moreover obtain some control on the rate at which the critical points escape. In fact, we will prove the existence of a subset $E \subset \mathbb C_w$ of full measure for which *all critical points* of maps $f_{w_n}$, for $w \in E$, escape to *W* at least at some moderate rate, while *most critical points* escape very fast.

The critical points of *F* form a finite union of irreducible varieties in $\mathbb {C}^2$, as described in Sect. 3. We will study these varieties one at a time. Given a fixed open neighborhood $U\subseteq \mathbb {C}$ of the post-critical set of the polynomial $p(\cdot ) = f(\cdot ,0)$, we will prove the following:

### Proposition 14

Let *K* be an irreducible critical variety. Then there exists a set $E_K\subseteq \mathbb {C}$, of full measure in some neighborhood of the origin, with the following property:

For all $\eta >0$ and $w \in E_K$ we have

$$\begin{aligned} \#\left\{ s \le n:~ \exists (z,\lambda ^s w)\in K \mathrm {\, with \,} F_1^{n-s}(z, \lambda ^s w) \notin W_0 \cup U \right\} \le \eta \log n +C_{4}, \end{aligned}$$

where $C_{4}$ is a constant depending on $K, \eta , U$, and *w*.

A consequence is the main result of this section.

### Proposition 15

There exists a set $E\subset \mathbb {C}$, of full measure in some neighborhood of the origin, with the following property: For all $w \in E$ there exists a constant $C_2(w,U)$ such that for all $n \in \mathbb {N}$ we have

$$\begin{aligned} \#\left\{ z:~ \frac{\partial F_1^n}{\partial z}(z,w)=0 \mathrm {\; and \;} F_1^n(z,w) \notin W_0 \cap U \right\} \le C_2\sqrt{n}. \end{aligned}$$

### Proof

Define $E=\bigcap _{K}E_K$, let $w \in E$ and let $\eta >0$. Pick $\epsilon '>0$ such that *E* has full measure in $D(0,\epsilon ')$. We have at most finitely many critical varieties: $K_1$ up to $K_q$. Applying Proposition 14 for each of them gives us

$$\begin{aligned} \#\left\{ s \le n:~ \exists (z,\lambda ^s w) \in \bigcup K_i \mathrm {\,with\,} F_1^{n-s}(z, \lambda ^s w) \notin W_0 \cup U \right\} \le q\eta \log n +C, \end{aligned}$$

where *C* depends only on $\eta , U$ and *w*. As $f_w(z)=z^d+a_{d-1}(w)z^{d-1}+\cdots +a_0(w)$, each $\{\lambda ^s w\}$-fiber contains $d-1$ critical points of $f_w(z)$, counting with multiplicities. Then the restricted function

$$\begin{aligned} F_1^n :(F^n)^{-1}\bigl (\left( \mathbb {C} \setminus (W_0 \cup U)\right) \times \{\lambda ^n w\} \bigr ) \rightarrow \left( \mathbb {C} \setminus (W_0 \cup U)\right) \end{aligned}$$

is a composition of *n* holomorphic functions. At most $q\eta \log n +C$ of them can each have at most $d-1$ critical points. The total number of critical points of this restricted function is therefore at most

$$\begin{aligned} d^{q\eta \log n +C}=d^{C}n^{q\eta \log d}. \end{aligned}$$

The desired result is obtained by choosing $\eta$ small enough. $\square$

The rest of this section is devoted to proving Proposition 14. This proposition will be proved in two parts. First, we will prove that for almost every $w_0 \in {\mathbb {C}}$ and all $n\in {\mathbb {N}}$, all critical points of $f_{w_n}$ escape to the set *W* in at most $C \cdot \log (n)$ steps, for some uniform $C>0$. We will not be able to control the constant *C* though, and hence we will not yet be able to deduce that the estimate in Proposition 14 holds for *any* $\eta >0$. However, we will also prove that for almost every $w_0 \in {\mathbb {C}}$ and *most* $n \in {\mathbb {N}}$, the critical points of $f_{w_n}$ escape to the set *W* in a number of steps that does not depend on *n*. By choosing a sufficiently strong interpretation of “most $n \in {\mathbb {N}}$” it will follow that $\eta$ in Proposition 14 can be chosen arbitrarily small.

Let *K* be one of the irreducible analytic varieties of critical points of *F* in $\mathbb {C} \times D(0,\epsilon )$, as described in Remark 7. Then *K* intersects the $\{w=0\}$-fiber in a single point $x_0 \in \mathbb {C}_z$. If $x_0 \in \mathcal {F}_p$, then $F^r(x_0) \in W_0$ for some $r \in \mathbb {N}$. Shrinking $\epsilon$ if necessary, we find that $F^r(K) \subseteq W$. In this setting, Proposition 14 follows immediately. We will therefore assume that $x_0 \in \mathcal {J}_p \times \{0\}$ from now on. Its image $x_r=p^r(x_0)$ is a repelling fixed point. We define $\mu =p'(x_r)$.

Since *K* intersects $\{w=0\}$ in a unique point, we can locally parameterize *K* by

$$\begin{aligned} K = \left\{ \bigl ( \gamma _1(u),\gamma _2(u)\bigr ): u \in D\bigl (0,\epsilon ^{1/l}\bigr )\right\} , \end{aligned}$$

where $\gamma _2(t)=t^l$ for some $l \ge 1$.

If $F^r(K)$ is contained in the stable variety $\Sigma _F^s(x_r)$ of *F* at $x_r$, then so is $F^n(K)$ for any $n \ge r$. The points in *K* all converge to $(x_r,0)$ along this stable variety. In this setting, Proposition 14 follows immediately.

For the rest of this section, we will therefore assume that $F^r(K) \nsubseteq \Sigma _F^s(x_r)$. Remark 7 then gives us the linearization maps

$$\begin{aligned} \varPhi _j(t)=F^{kj}\left( \gamma _1(\mu ^{-j}t),(\mu ^{-j}t)^l \right) \end{aligned}$$

that converge uniformly and exponentially fast on compact subsets, to a holomorphic function $\varPhi :\mathbb {C} \rightarrow \mathbb {C}_z$ of local degree *k* for some $k \ge 1$.

We wish to study the points of the critical variety *K* in a sequence of fibers $\mathbb {C} \times \{w\}, \mathbb {C} \times \{\lambda w\}, \mathbb {C} \times \{\lambda ^2 w\}, \ldots$ where $w \in D(0,\epsilon )$. This will be easier if we look at pre-images of these critical points under $\gamma$: pick $\nu \in \mathbb {C}$ such that $\nu ^l=\lambda$ and consider the sequences $u, \nu u, \nu ^2 u \ldots$ for $u \in D(0,\epsilon ^{1/l})$. By abuse of notation, we replace the constant $\epsilon ^{1/l}$ by the letter $\epsilon$ again. For $u \in D(0,\epsilon )$, the orbit of $\gamma (\nu ^s u)$ under *F* can now be studied using the functions $\varPhi _j$ and $\varPhi$:

$$\begin{aligned} F^n\bigl (\gamma (\nu ^s u)\bigr )&=F^{n-kj}\left( \varPhi _j(\mu ^j \nu ^s u)\right) \text { whenever } n \ge kj\\&\approx F^{n-kj}\left( \varPhi (\mu ^j \nu ^s u)\right) . \end{aligned}$$

In order to use exponential estimates on the rate of convergence of $\varPhi _j$ to $\varPhi$, we ask that $|\mu ^j \nu ^s u| \le \epsilon$, while having *j* run to infinity. We therefore define *j*(*s*) to be the unique integer for which

$$\begin{aligned} |\mu |^{-1}<|\mu ^{j(s)} \nu ^s| \le 1. \end{aligned}$$

Lemma 3 now implies the following:

### Corollary 16

There exist constants $C_5, C_6 > 0$ such that for all $s,m \in \mathbb {N}$ satisfying $m < C_5j(s)-C_6$ and for all $u \in D(0,\epsilon )$, we have

$$\begin{aligned} \text {If }\varPhi \bigl (\mu ^{j(s)} \nu ^s u\bigr ) \in U_m, \quad \mathrm{then }\ \ F^{m+1+kj(s)}\bigl (\gamma (\nu ^s u)\bigr ) \in W. \end{aligned}$$

### Proof

If $\varPhi (\mu ^{j(s)} \nu ^s u) \in U_m$ and *j*(*s*) is large enough to ensure that $||\varPhi _{j(s)}-\varPhi ||_{D(0,\epsilon )} \le C_1 M^{-m}$, then $F^{m+1+kj(s)}(\gamma (\nu ^s u))=F^{m+1}(\varPhi _{j(s)}(\mu ^{j(s)}\nu ^s u)) \in W$ by Lemma 3. By the uniform convergence of $\varPhi _j$ to $\varPhi$ on $D(0,\epsilon )$, the requirement that $||\varPhi _{j(s)}-\varPhi ||_{D(0,\epsilon )} \le C_1 M^{-m}$ can be translated to $m<C_5j(s)-C_6$ for some uniform constants $C_5$ and $C_6$. $\square$

Corollary 16 motivates us to take a closer look at $(\varPhi |_{D(0,\epsilon )})^{-1}(U_m)$.

### All Critical Points Escape Slowly

The complement of $(\varPhi |_{D(0,\epsilon )})^{-1}(U_m)$ within $D(0,\epsilon )$ is exactly $(\varPhi |_{D(0,\epsilon )})^{-1}(V_m)$. By Proposition 10 the area of $V_m$ decreases exponentially with *m*. As $\varPhi$ is a non-constant holomorphic function, the area of $\left( \varPhi |_{D(0,\epsilon )}\right) ^{-1}(V_m)$ also decreases exponentially with *m*, although not necessarily at the same rate.

Using this information, we can apply Corollary 16 to show that given $s \in \mathbb {N}$, the critical point $\gamma (\nu ^s u)$ moves away from the Julia set $\mathcal {J}_p$ fairly quickly for most values $u \in D(0,\epsilon )$.

#### Lemma 17

There exist uniform constants $C_7>0$ and $\alpha <1$ such that

$$\begin{aligned} \text {Area}\left\{ u \in D(0,\epsilon ) ~:~ F^{kj(s)+m+1}\bigl (\gamma (\nu ^s u)\bigr ) \notin W \right\} \le C_7 \alpha ^m \end{aligned}$$

for all $s,m \in \mathbb {N}$ with $m<C_5j(s)-C_6$.

#### Proof

Corollary 16 implies the inclusion

$$\begin{aligned} \left\{ u \in D(0,\epsilon ) ~:~ F^{kj(s)+m+1}\bigl (\gamma (\nu ^s u)\bigr ) \notin W \right\}&\subseteq \left\{ u \in D(0,\epsilon ) ~:~ \varPhi \bigl (\mu ^{j(s)} \nu ^s u\bigr ) \notin U_m\right\} \\&= D(0,\epsilon ) \cap \mu ^{-j(s)}\nu ^{-s} \varPhi ^{-1}(V_m)\\&\subseteq \mu ^{-j(s)}\nu ^{-s} \left( \varPhi |_{D(0,\epsilon )}\right) ^{-1}(V_m). \end{aligned}$$

The area of this last set is at most $|\mu |^{-1} \text {Area}((\varPhi |_{D(0,\epsilon )})^{-1}(V_m))$, which decreases exponentially with *m*. $\square$

We will use Lemma 17 to find values of $u\in D(0,\epsilon )$ for which nearly all critical points $\{\gamma (\nu ^s u)\}_{s \in \mathbb {N}}$ move away from $\mathcal {J}_p$ fairly fast. Our strategy is to apply the following well-known lemma:

#### Lemma 18

Let $\{A_\mathrm{s}\}_{n \in \mathbb {N}}$ be a sequence of subsets of a measure space *X* such that

$$\begin{aligned} \sum _{s \in N} \mathrm{measure}(A_\mathrm{s}) < \infty . \end{aligned}$$

Then the set $E=\{u \in X ~|~ \exists N_u \in \mathbb {N},~ \forall s \ge N_u,~ u \notin A_\mathrm{s} \}$ has full measure.

To be able to use Lemma 17 in the setting of Lemma 18, we need to pick an appropriate value *m*(*s*) for each $s \in \mathbb {N}$. Then we can apply Lemma 18 to the sets

$$\begin{aligned} A_\mathrm{s}=\left\{ u \in D(0,\epsilon ) ~:~ F^{kj(s)+m(s)+1}\bigl (\gamma (\nu ^s u)\bigr ) \notin W \right\} . \end{aligned}$$

To make sure that the areas of the sets $A_\mathrm{s}$ have a finite sum, we need to pick *m*(*s*) such that

$$\begin{aligned} \sum _{s \in \mathbb {N}} C_7 \alpha ^{m(s)} < \infty . \end{aligned}$$

On the other hand, it would be preferable to pick *m*(*s*) as low as possible. This gives stronger information on the rate at which a critical point $\gamma (\nu ^s u)$ (for $u \notin A_\mathrm{s}$) moves towards *W*. And finally, *m*(*s*) needs to satisfy $m(s)<C_5j(s)-C_6$ to be allowed to use the estimate in Lemma 17.

To satisfy all these requirements, we define

$$\begin{aligned} m(s) := \left\lceil \frac{-2 \log s}{\log \alpha } -C_8 \right\rceil , \end{aligned}$$

where the constant $C_8>0$ is chosen such that $m(s)<C_5j(s)-C_6$ holds for all $s \in \mathbb {N}$. As $\alpha ^{m(s)}\le \alpha ^{-C_8} s^{-2}$, the areas of the sets $A_\mathrm{s}$ will have their finite sum. We have now proved the following corollary:

#### Corollary 19

The set

$$\begin{aligned} E_1(K)=\left\{ u \in D(0,\epsilon )~\Big |~ \exists N_u \in \mathbb {N},~ \forall s \ge N_u,~ F^{kj(s)+m(s)+1}\bigl (\gamma (\nu ^s u)\bigr ) \in W \right\} \end{aligned}$$

has full measure in $D(0,\epsilon )$.

For points $\nu ^s u$ with $u \in E_1(K)$ and $s \ge N_u$, we now have a reasonable grasp of the behavior of $F^n(\gamma (\nu ^s u))$ when $n \ge kj(s)$: the point will move to *W* in at most another $N+m(s)$ applications of the function *F*. The next lemma will take a look at the setting when $n<kj(s)$. For any neighborhood $U\subseteq \mathbb {C}$ of the post-critical set of *p*, we have the following:

#### Lemma 20

For $u \in D(0,\epsilon )$ we have

$$\begin{aligned} F_1^n\bigl (\gamma (\nu ^s u)\bigr ) \in U \end{aligned}$$

whenever $C_9 \le n < kj(s)-C_9$, for a constant $C_9(\epsilon , U)$ that is independent of *s*.

#### Proof

Pick $\rho >0$ such that $D(x_r,\rho ) \subseteq U \cap D(0,R)$. Fix $\theta$ such that $\varPhi (D(0,\theta )) \subseteq D(x_r, M^{-k}\rho /2)$. Write $n=ki+v$ with $0 \le v <k$, and note that

$$\begin{aligned} F^n\bigl (\gamma (\nu ^s u)\bigr )=F^v\bigl (\varPhi _i(\mu ^i \nu ^s u)\bigr ). \end{aligned}$$

Assume that *i* is small enough to have $|\mu ^i \nu ^s \epsilon |<\theta$. On the other hand, assume that *i* is large enough to ensure that $||\varPhi _i - \varPhi ||_{D(0,\epsilon )} < M^{-k}\rho /2$. Then for $u \in D(0,\epsilon )$ we have $\text {d}\left( \varPhi _i(\mu ^i \nu ^s u),x_r\right) < M^{-k}\rho$. As $x_r$ is a fixed point of *F* and $v<k$, this implies that $\text {d}\left( F^v(\varPhi _i(\mu ^i \nu ^s u)),x_r\right) < \rho$ and hence $F^n(\gamma (\nu ^s u)) \in U$. Here, we used the fact that $D(x_r,\rho ) \subseteq D(0,R)$ and that *M* is an upper bound for |*DF*| on $D(0,R) \times D(0,\epsilon )$.

As $|\mu ^{j(s)}\nu ^s|<1$, the requirements on *i* can be translated to $C_9 \le n < kj(s)-C_9$ for some constant $C_9(\epsilon ,U)$. $\square$

Combining Corollary 19 with Lemma 20 allows us to prove the slow escape of the points in the critical variety *K*:

#### Proposition 21

For any $u \in E_1(K)$ and $n \in \mathbb {N}$, we have

$$\begin{aligned} \#\left\{ s \le n:~ F_1^{n-s}\bigl (\gamma (\nu ^s u)\bigr ) \notin W_0 \cup U \right\} \le C_{10} \log n +C_{11}, \end{aligned}$$

for constants $C_{10}(u,K,U,\epsilon )$ and $C_{11}(u,K,U,\epsilon )$ independent of *n*.

#### Proof

Write $S_n=\left\{ s \le n:~ F_1^{n-s}(\gamma (\nu ^s u)) \notin W_0 \cup U \right\}$. By definition of $E_1(K)$ and the fact that $F(W) \subseteq W$, we know that $s \notin S_n$ when *s* satisfies both $s \ge N_u$ and $n-s \ge kj(s)+m(s)+1$.

By Lemma 20, we know that $s \notin S_n$ when $C_9 \le n-s < kj(s)-C_9$. This leaves us with $2C_9+N_u$ possible entries in $S_n$, plus all values *s* for which

$$\begin{aligned} kj(s) \le n-s <kj(s)+m(s)+1. \end{aligned}$$

Such a value of *s* must also satisfy

$$\begin{aligned} n&\ge s+kj(s)&>n-m(n)-1,&\text { and hence}\\ n&\ge s+k\left\lfloor \frac{s}{l} \frac{\log |\lambda ^{-1}|}{\log |\mu |} \right\rfloor&>n-m(n)-1,&\text { and }\\ n+k&\ge s\left( 1+\frac{k}{l} \frac{\log |\lambda ^{-1}|}{\log |\mu |}\right)&>n-m(n)-1.&\end{aligned}$$

Plugging in our formula for *m*(*n*) shows that this gives us a sequence of at most

$$\begin{aligned} \frac{k+1+\left\lceil \frac{-2 \log n}{\log \alpha } -C_8 \right\rceil }{1+\frac{k}{l} \frac{\log |\lambda ^{-1}|}{\log |\mu |}} \end{aligned}$$

consecutive values of *s*. This proves the proposition. $\square$

### Most Critical Points Escape Quickly

Proposition 21 and its proof showed that for each $u \in E_1(K)$ and $n \in \mathbb {N}$, the set

$$\begin{aligned} S_n(u)=\left\{ s \le n:~ F_1^{n-s}\bigl (\gamma (\nu ^s u)\bigr ) \notin W_0 \cup U \right\} \end{aligned}$$

could contain at most $C_{11}$ points, plus possibly some integers in an interval of length $C_{10} \log n$. Very little information about the dynamical system was used to prove this. In this subsection, we will show that for almost all $u \in E_1(K)$, an arbitrarily large portion of the values *s* in any sufficiently large interval do not lie in $S_n$.

Recall that *j*(*s*) is the unique natural number such that

$$\begin{aligned} |\mu ^{-1}|<|\mu ^{j(s)} \nu ^s |\le 1. \end{aligned}$$

The sequence $\{\mu ^{j(s)} \nu ^s\}_{s \in \mathbb {N}}$ lives in the annulus $\{|\mu ^{-1}|<|t| \le 1\}$. Multiplication by $\mu$ allows us to identify the inner and outer boundaries of the annulus, giving us the torus $\left( \mathbb {C} \setminus \{0\}\right) /\mu ^{\mathbb {Z}}$. It will be more convenient to think about this torus additively. Write

$$\begin{aligned} T=\mathbb {C} / (2\pi i \mathbb {Z}+\log \mu \mathbb {Z}). \end{aligned}$$

It does not matter which value for $\log \mu$ we use, since $2 \pi i$ is included in our lattice anyway. As $|\mu |>1$, we know that $\log \mu$ is not purely imaginary. Composing a projection with any branch of the logarithm, we obtain a holomorphic covering map:

$$\begin{aligned} \varPsi :\quad \mathbb {C} \setminus \{0\} \mathop {\longrightarrow }\limits ^{\log } \mathbb {C} \mathop {\longrightarrow }\limits ^{\pi } \mathbb {C} / (2\pi i \mathbb {Z}+\log \mu \mathbb {Z})=T. \end{aligned}$$

The projection $\pi$ ensures that the branch of the logarithm is irrelevant. When restricted to $\{|\mu ^{-1}|<|t| \le 1\}$, the map $\varPsi$ is a bijection.

Let *y* be any value for $\log \nu$. Then in the torus *T*, the sequence $\{\varPsi (\mu ^{j(s)} \nu ^s)\}_{s \in \mathbb {N}}$ is represented by $\{s y\}_{s \in \mathbb {N}}$. We recall the following lemma from Sect. 1.4 of [5].

#### Lemma 22

Let *T* be an additive torus and $y \in T$. Then the set $\overline{\{s y\}_{s \in \mathbb {N}}}$ is a subgroup of *T*, and one of the following statements must be true:

1. The set $\overline{\{s y\}_{s \in \mathbb {N}}}$ is finite.
2. The set $\overline{\{s y\}_{s \in \mathbb {N}}}$ is one-dimensional. It is a disjoint union of finitely many evenly spaced lines/circles in *T*.
3. The set $\overline{\{s y\}_{s \in \mathbb {N}}}$ equals *T*.

The torus *T* can be covered by cosets $[a]=a+\overline{\{s y\}_{s \in \mathbb {N}}}$ for $a \in T$. Denote by $\sigma _a$ the counting measure, the one-dimensional Lebesgue measure, or the two-dimensional Lebesgue measure on [*a*] for cases (1), (2), or (3), respectively. The action of $x \mapsto x+y$ on [*a*] is uniquely ergodic, and hence we have

#### Lemma 23

Let $\eta >0, a \in T$, and $O \subseteq [a]$ open. Then there exists $C_{12}(\eta ,O,T)>0$ such that for all $r \in \mathbb {N}$ and $n \ge C_{12}$ we have

$$\begin{aligned} \frac{1}{n} \# \left\{ r < s \le r+n: a+sy \in O \right\} > \frac{\sigma _a(O)}{\sigma _a([a])}-\eta . \end{aligned}$$

See for example Sect. 4.3 in [3].

Since $\mathcal {J}_p$ is *F*-invariant and $\varPhi$ satisfies the equation $F^k \circ \varPhi (t) = \varPhi (\mu t)$, the set $\varPhi ^{-1}(\mathcal {J}_p)$ must be invariant under multiplication by $\mu$. By our assumptions $\mathcal {J}_p$ has measure zero, so since $\varPhi$ is a non-constant holomorphic function, the set $\varPhi ^{-1}(\mathcal {J}_p)\cap D(0,1)$ must also have measure zero. Note that $\varPhi ^{-1}(\mathcal {J}_p)$ is also closed. It follows that $\varPsi \left( \varPhi ^{-1}(\mathcal {J}_p)\right)$ is a compact set of measure zero.

For any $a \in T$, the set $\varPsi \left( \varPhi ^{-1}(\mathcal {J}_p)\right) \cap [a]$ will be a closed subset. By Fubini’s Theorem, it follows that $\sigma _a\left( \varPsi \left( \varPhi ^{-1}(\mathcal {J}_p)\right) \cap [a]\right) =0$ for almost every $a \in T$. Denote the set of these values of *a* by *A*.

Let $\eta >0$. For each $a \in A$, we can choose an open set $O(a,\eta )\subseteq [a]$ such that $\sigma _a(O) \ge (1-\eta )\sigma _a([a])$ and ${\overline{O}} \cap \varPsi \left( \varPhi ^{-1}(\mathcal {J}_p)\right) =\emptyset$. By Lemma 23 we can find $C_{12}(a,\eta )$ such that

$$\begin{aligned} \frac{1}{n}\# \left\{ r<s \le r+n: a+sy \in O \right\} > 1-2\eta \quad \text{ for } \text{ all } r\in \mathbb {N},n\ge C_{12}. \end{aligned}$$

Define $E_2(K)=\{u \in D(0,\epsilon ):u\ne 0 \text { and } \varPsi (u) \in A\}$. As *A* has full measure in *T*, the set $E_2(K)$ has full measure in $D(0,\epsilon )$. For any $u \in E_2(K), r \in \mathbb {N}$, and $n\ge C_{12}$ we can translate the above statement to

$$\begin{aligned} \frac{1}{n}\# \left\{ r<s \le r+n: \mu ^{j(s)}\lambda ^s u \in \varPsi ^{-1}({\overline{O}})\cap \{|\mu ^{-1}u| \le |t| \le |u|\} \right\} > 1-2\eta . \end{aligned}$$

The set $\varPsi ^{-1}({\overline{O}})\cap \{|\mu ^{-1}u| \le |t| \le |u|\}$ is closed and bounded, hence compact. Its intersection with $\varPhi ^{-1}(\mathcal {J}_p)$ is empty. We can therefore cover $\varPsi ^{-1}({\overline{O}})\cap \{|\mu ^{-1}u| \le |t| \le |u|\}$ by the increasing open sets $\{\varPhi ^{-1}(U_m)\}_{m \in \mathbb {N}}$. By compactness, we can find $\ell =\ell (u,\eta )$ such that $\varPsi ^{-1}({\overline{O}})\cap \{|\mu ^{-1}u| \le |t| \le |u|\}\subseteq \varPhi ^{-1}(U_\ell )$.

We can now prove the following proposition:

#### Proposition 24

For all $\eta >0$ and $u \in E_2(K)$, we have

$$\begin{aligned} \frac{1}{n}\# \left\{ r<s \le r+n: F^{kj(s)+\ell +1}\bigl (\gamma (\lambda ^s u)\bigr ) \in W \right\} > 1-2\eta , \end{aligned}$$

whenever $n \ge C_{12}$ and $r \ge C_{13}$. Here $\ell =\ell (u,\eta )$ and $C_{12}(u,\eta )$ are as defined above and $C_{13}$ depends on $\ell$.

#### Proof

Let $\eta >0, u \in E_2(K)$ and $n \ge C_{12}(u,\eta )$. We start by the observation that

$$\begin{aligned} 1-2\eta&< \frac{1}{n}\# \left\{ r<s \le r+n: \mu ^{j(s)}\lambda ^s u \in \varPhi ^{-1}(U_\ell ) \right\} \\&=\frac{1}{n}\# \left\{ r<s \le r+n: \varPhi (\mu ^{j(s)}\lambda ^s u) \in U_\ell \right\} \end{aligned}$$

for all $r \in \mathbb {N}$. When *r* is sufficiently large, *j*(*s*) will be large enough to ensure that the latter set is included in

$$\begin{aligned} \frac{1}{n}\# \left\{ r<s \le r+n: d\left( \varPhi _{j(s)}(\mu ^{j(s)}\lambda ^s u), U_\ell \right) <C_1M^{-\ell } \right\} , \end{aligned}$$

and by Lemma 3, this set is contained in the set

$$\begin{aligned} \frac{1}{n}\# \left\{ r<s \le r+n: F^{\ell +1}\left( \varPhi _{j(s)}(\mu ^{j(s)}\lambda ^s u)\right) \in W \right\} > 1-2\eta . \end{aligned}$$

Combining all this with the definition of $\varPhi _{j(s)}$ proves the proposition. $\square$

Define $E_3(K)=E_1(K) \cap E_2(K)$. This set still has full measure in $D(0,\epsilon )$, and we can combine Propositions 21 and 24 to find

#### Corollary 25

Let $\eta >0$ and $u \in E_3(K)$. Then there exists a constant $C_{14}$, depending on $\eta , u, U, \epsilon$, and *K*, such that for all $n \in \mathbb {N}$ we have

$$\begin{aligned} \#\left\{ s \le n:~ F_1^{n-s}\bigl (\gamma (\nu ^s u)\bigr ) \notin W_0 \cup U \right\} \le \eta \log n +C_{14}. \end{aligned}$$

#### Proof

As in the proof of Proposition 21, write

$$\begin{aligned} S_n=\left\{ s \le n:~ F_1^{n-s}\bigl (\gamma (\nu ^s u)\bigr ) \notin W_0 \cup U \right\} . \end{aligned}$$

We showed that $S_n$ contains $2C_9+N_u$ entries, plus possibly the values *s* for which

$$\begin{aligned} kj(s) \le n-s <kj(s)+m(n)+1. \end{aligned}$$

The length of this interval was bounded by $C_{10} \log n$, for a constant $C_{10}$ depending on *u*, *K*, *U*, and $\epsilon$. Choose $\theta >0$ such that $2 \theta C_{10} < \eta$ and apply Proposition 24:

Whenever $n-s \ge kj(s)+\ell +1$ and $s \ge C_{13}$, no interval of length $L \ge C_{12}$ will contain more than $2\theta L$ elements of $S_n$. We can now pick $C_{14}$ to be large enough to ensure that $\#S_n \le \eta \log n +C_{14}$ for all $n \in \mathbb {N}$. $\square$

#### Proof of Proposition 14

Write $Q=D(0,\epsilon ) \setminus E_3(K)$, a set of measure zero. The set $Q^l=\{u^l : u \in Q\}$ still has measure zero. Define $E_K=D(0,\epsilon ^l) \setminus Q^l$. Then for each $w \in E_K$ we have

$$\begin{aligned}&\#\left\{ s \le n:~ \exists (z,\lambda ^s w)\in K \text { with }F_1^{n-s}(z, \lambda ^s w) \notin W_0 \cup U \right\} \\&\quad =\, \bigcup _{u: \; u^l=w} \#\left\{ s \le n:~ F_1^{n-s}\bigl (\gamma (\nu ^s u)\bigr ) \notin W_0 \cup U \right\} \le l\eta \log n +C_4. \end{aligned}$$

for a constant $$C_4=C_4(K,\eta ,U,w)$$. $$\square $$

## Hyperbolic Areas of Inverse Images

Only Proposition 28 remains to be shown. First recall the following similar result from [9].

### Lemma 26

For any $0<r<1$ and any holomorphic proper map $f:V \rightarrow {\mathbb {D}}$ of degree *d*, with *V* simply connected, each connected component of $f^{-1}(\overline{D_r}(0))$ has diameter at most

$$\begin{aligned} 2d\log \left( \frac{1+r^{1/d}}{1-r^{1/d}}\right) \end{aligned}$$

with respect to the Poincaré metric on *V*.

Our goal is to prove a similar estimate regarding hyperbolic areas rather than diameters. Our strategy will be to remove small neighborhoods of critical points, and prove that the area of the inverse image outside of these neighborhoods must be small. We first give an estimate on the proximity of a critical point for points with small derivatives.

### Lemma 27

If $f(z) :\mathbb {D} \rightarrow \mathbb {D}$ is a proper holomorphic map of degree *d* satisfying $f(0)=0$ and $|f'(0)|\le \delta ^{d-1}$ with $\delta <1/4$, then *f* has a critical point *c* with $d_{\mathbb {D}}(c,0)\le 32d\delta$.

### Proof

Without loss of generality may write

$$\begin{aligned} f(z)= z\prod _{i=1}^{d-1}\frac{z-a_i}{1-{\bar{a}}_iz}. \end{aligned}$$

We may assume that $|a_1| \le |a_j|$ for $j \ge 2$. By our assumption that $\delta ^{d-1} \ge |f'(0)|=|\prod _{i=1}^{d-1}a_i|$, it follows that $|a_1|<\delta$. For $z=ta_1$ with $t \in [0,1]$ we have

$$\begin{aligned} f(z)&=z\prod _{i=1}^{d-1}\frac{z-a_i}{1-{\bar{a}}_iz}=z(z-a_1)\prod _{i=2}^{d-1}(z-a_i) \prod _{i=1}^{d-1}\frac{1}{1-{\bar{a}}_iz}, \; \text { and hence}\\ |f(z)|&\le \frac{|a_1|^2}{4}\prod _{i=2}^{d-1}(2|a_i|)\prod _{i=1}^{d-1}\frac{1}{1-\delta } \le \left( \frac{2\delta }{1-\delta }\right) ^d. \end{aligned}$$

Let $r= (\frac{2\delta }{1-\delta })^d$. By the above estimate the set $f^{-1}\left( D(0,r)\right)$ contains the interval between 0 and $a_1$. As both endpoints of this interval are mapped to 0 and *D*(0, *r*) is simply connected, the connected component of $f^{-1}\left( D(0,r)\right)$ that contains this interval must also contain a critical point *c*. By Lemma 26 the Poincaré distance from *c* to 0 can therefore be estimated by

$$\begin{aligned} 2d\log \left( \frac{1+r^{1/d}}{1-r^{1/d}}\right) =2d\log \left( \frac{1+\frac{2\delta }{1-\delta }}{1-\frac{2\delta }{1-\delta }}\right) \le 2d \cdot 4 \frac{2\delta }{1-\delta }\le 32d\delta \end{aligned}$$

For the first inequality, we used our assumption that $\delta < 1/4$. $\square$

Let $f:{\mathbb {D}} \rightarrow {\mathbb {D}}$ be a proper holomorphic map of degree *d*. Suppose that $x \in \mathbb {D}$ satisfies $d_{\mathbb {D}}(x,c_i) > 32d\delta$ for each critical point $c_i$. Write $f(x)=y$ and define the functions $g(z)=\frac{z+x}{1+{\bar{x}}z}$ and $h(z)=\frac{z-y}{1-{\bar{y}}z}$. Since *g* and *h* preserve the Poincaré metric, the point $0=g^{-1}(x)$ has Poincaré distance at least $32d\delta$ to any critical point of $h\circ f \circ g$, and Lemma 27 gives

$$\begin{aligned} \delta ^{d-1} \le |(h\circ f \circ g)'(0)|=|h'(y)||f'(x)||g'(0)|=\frac{1-|x|^2}{1-|y|^2}|f'(x)|. \end{aligned}$$

### Proposition 28

Let $f:{\mathbb {D}} \rightarrow {\mathbb {D}}$ be a proper holomorphic map of degree *d*, and let $R \subseteq \mathbb {D}$ have Poincaré area *A*. If $d\cdot A^{1/2d}<1/8$, the inverse image of *R* under *f* will have Poincaré area at most $C_3d^3 A^{1/d}$ for a uniform constant $C_3$.

### Proof

Define $\delta =A^{1/2d}$ and let $S=\{z\in \mathbb {D}~|~ d_{\mathbb {D}}(z,c_i)>32d\delta \text { for all } i \}$. Then $f^{-1}(R) \subseteq f|_S^{-1}(R) \cup (\mathbb {D} \setminus S)$ and we can estimate the areas of these sets separately.

To estimate the area of $\mathbb {D} \setminus S$, note that the hyperbolic area of a disk of hyperbolic radius *s* is equal to $4\pi \sinh ^2(s/2)$, which is smaller than $4\pi s^2$ whenever $s<4$. As $\mathbb {D}\setminus S$ is a union of $d-1$ disks of hyperbolic radius $32d\delta <4$, we find

$$\begin{aligned} \text {Area}_{\mathbb {D}}(\mathbb {D}\setminus S) \le (d-1)4\pi 2^{10} d^2 \delta ^2<2^{12} \pi d^3 \delta ^2. \end{aligned}$$

To estimate $f|_S^{-1}(R)$, we use our estimate on the derivative. For any open set $U \subseteq f|_S^{-1}(R)$ such that $f|_U$ is conformal, we find

$$\begin{aligned} \text {Area}_{\mathbb {D}}(f(U))&=\iint \nolimits _{f(U)}\frac{4}{(1-|w|^2)^2}\,d\lambda (w)\\&=\iint \nolimits _U \frac{4}{(1-|f(z)|^2)^2}|f'(z)|^2\,d\lambda (z) \\&\ge \iint \nolimits _U \frac{4}{(1-|f(z)|^2)^2}\frac{(1-|f(z)|^2)^2}{(1-|z|^2)^2}\delta ^{2d-2}\,d\lambda (z)=\delta ^{2d-2} \text {Area}_{\mathbb {D}}(U). \end{aligned}$$

Since *f* has topological degree *d*, we can therefore estimate the Poincaré area of $f|_S^{-1}(R)$ by $\frac{d}{\delta ^{2d-2}} \cdot A=d\delta ^2$. Combining this with our estimate on the area of $\mathbb {D} \setminus S$ gives

$$\begin{aligned} \text {Area}_{\mathbb {D}}(f^{-1}(R))\le d \delta ^2+2^{12} \pi d^3\delta ^2 \le C_3 d^3 A^{1/d}. \end{aligned}$$

$$\square $$
